# Theoretical Analysis of Airy–Gauss Bullets Obtained by Means of High Aperture Binary Micro Zonal Plate

**DOI:** 10.3390/mi13020279

**Published:** 2022-02-10

**Authors:** Salvador Blaya, Edmundo Lopez-Sola, Pablo Acebal, Luis Carretero

**Affiliations:** Departamento de Ciencia de Materiales, Óptica y Tecnología Electrónica, Universidad Miguel Hernández de Elche, Av. de la Universidad s/n, 03202 Elche, Spain; edmundo.lopez@neuroelectrics.com (E.L.-S.); pacebal@umh.es (P.A.); l.carretero@umh.es (L.C.)

**Keywords:** Airy beams, micro zonal plates, non-diffracting optical bullets

## Abstract

We theoretically analyze the methodology for obtaining vectorial three-dimensional bullets, concretely Airy–Gauss bullets. To do this, binary micro zonal plates (BZP) were designed in order to obtain different Airy–Gauss bullets with sub-diffraction main lobe width. Following the vectorial diffraction theory, among the electrical field, we extend the theory to the magnetic field, and thus we analyze several properties such as the Poynting vector and the energy of Airy–Gauss vectorial bullets generated by illuminating the designed BZP with a temporal Gaussian circular polarized pulses.

## 1. Introduction

In recent years, due to their nondiffracting, self-accelerating, and self-healing properties, airy beams have attracted interest in linear and non-linear optics fields [1]. Currently, Airy beams are experimentally available, and, as a result, a wide interest has been paid in optical beam manipulation applications. Thus, by using the particular properties of Airy beams, several applications have been developed such as Airy plasma guiding [2], routing surface plasmon polaritons [3], image signal transmission [4], optical micromanipulation [5], optical bullets [1,6], optical trapping [7,8], and biomedical applications [9,10,11].

Experimentally, Airy beams have been generated by imposing a cubic phase on a Gaussian beam and then taking its Fourier transform by using a lens [1,12,13]. Concretely, several methods have been used to do this; thus, spatial light modulators (SLM) have been employed for obtaining a cubic phase profile. However, the beam quality is limited due to the discrete behavior of the device resulting in low efficiency. In a similar manner, liquid crystals and polymeric liquid crystals have also been used, but two Airy beams are simultaneously generated in this case [14,15]. Among these, cubic phase zonal phase plates [16,17,18] have been designed for obtaining high focusing capability.

Lastly, plasmonic and dielectric metasurfaces have transformed photonics research. Metasurfaces are planar versions of metamaterials and have shown unprecedented ability to arbitrarily manipulate light wavefronts, providing efficient solutions to generate desired accelerating beams [19,20]. These artificially designed 2D structured can be made of arrays of sub-wavelength scatterers for arbitrary tuning the characteristics of electromagnetic waves such as the control of Airy beams. Due to the size of the metasurface unit being much less than the wavelength, the limitations of conventional optical devices have relieved. As a result these new devices produce an increase in the bandwidth, generation efficiency, and integration capacity [21,22,23].

In many applications, it is vital that the light beams sharply autofocus their intensity right before a focal point, while keeping a low intensity until that very moment. To this end, the Airy beams were evolved into autofocusing beams [24,25] using a radially Airy symmetric intensity distribution. Generation and propagation of the these circular Airy beams with the abruptly autofocusing property were widely investigated [26,27,28] due to its potential value in optical micro-manipulation [29,30,31,32], and the excellent property makes them especially beneficial for biomedical treatment, laser ablation, and the generation of high-intensity lasers [11,33,34].

Moreover, among the applications of the Airy beams, the interest in the generation of temporal and spatial localized light pulses (optical bullets) has raised in recent years. In this sense, the Airy functions bring the possibility, in a linear regime, to separate spatial and temporal variables to obtain the 3D spatio-temporal beam [13,35,36]. Following, the theoretical procedure proposed by Siviloglou [13], Chong experimentally demonstrated the generation of light bullets in a linear regime, to be exact a spatial Bessel and a temporal Airy [37,38,39], and Abdollahpour obtained the Airy–Airy bullets [6]. Furthermore, theoretically, by solving in paraxial conditions the Schrödinger equation, different non-diffracting beams systems have been described such as Airy-parabolic-cylindrical [40], Airy-Hermite-Gauss [41], and Airy-Tricomi-Gauss [42].

Recently, based on the diffraction theory, we analyzed the generation of vectorial Airy–Airy light bullets by means of binary micro zonal plates (BZP). Thus, we developed a theoretical methodology for obtaining and analyzing Airy–Airy vectorial bullets [43]. In this work, based on the previous results, we studied Airy–Gauss light bullets. To do this, we extended the theoretical analysis to the magnetic field, which gives the possibility of studying additional properties such as the Poynting vector, energy, and the helicity of the resulting Airy–Gauss bullets.

## 2. Theoretical Description

**Figure 1:**
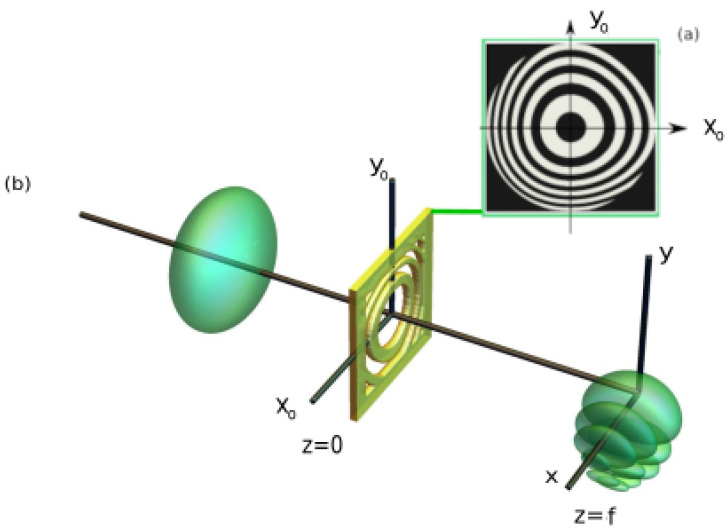
(**a**) Designed binary microplate of dimensions with parameters shown in Table 1. (**b**) Diagram for the generation of Airy–Gauss bullets, comprising intensity iso-surfaces of the incident Gaussian beam and the generated spatio-temporal (Airy–Gauss bullet) by the designed binary microplate.

As we have stated, Chong experimentally demonstrated the generation of light bullets in a linear regime, to be exact a spatial Bessel and temporal Airy [37,38,39]. The experimental setup, basically, can be divided in two parts (the temporal profile generation), and the second one is the spatial generation. In our case, instead of the case analyzed by Chong, in the first part (temporal profile generation), Gaussian temporal pulses were obtained from a mode-locked laser. In the second part, instead of an Axicon (to obtain a Bessel profile), the spatial Gaussian beam was converted into a spatial Airy beam by using a Binary Zone Plate. In this work we, theoretically, propose a BZP that could be used to obtain Airy–Gauss bullets. Applying the same methodology that we previously described [43], we design a binary zonal plate to focus real or complex transmittance $T(x_{o},y_{o})$ defined at the z=0 plane in a focal plane located at z=f (see Figure 1). It is important to mention that in this work, the BZP is not fabricated; only the theoretical function is proposed, and to encode it in a material, a directing laser writing technique could be used [17,44]. Furthermore, according to Figure 1, it is assumed that the medium in z>0 region is water, but air or other linear media can be used. The construction of zonal micro-plates with binary transmittance $T_{ZM}\left(x_{o},y_{o}\right)$ is based on the following function:

(1) $$T_{ZM}\left(x_{o},y_{o}\right)=\left(1-I(Mod\left(\psi \left(x_{o},y_{o}\right)/2\pi ,1\right))\right)$$

where I() and Mod() are the round function and module 1 operator, respectively. According to this definition, T${}_{ZM}$ will be a binary amplitude real function. Furthermore, the function $\psi (x_{o},y_{o})$ (Equation (1)) will simultaneously contain information about the transmittance of the binary zonal plate, $T(x_{o},y_{o})$, and a spherical convergent wave of wave number $k_{0}=\omega_{0}\;n/c$ that focuses at plane z=f according to [45]:

(2) $$\psi \left(x_{o},y_{o}\right)=Arg\left(T\left(x_{o},y_{o}\right)\;\frac{\exp\left(-i\;k_{0}\sqrt{x_{o}^{2}+y_{o}^{2}+f^{2}}\right)}{\sqrt{x_{o}^{2}+y_{o}^{2}+f^{2}}}\right)$$

In this work, to generate a spatial Airy beam, the designed zonal plates must fulfill that $T\left(x_{o},y_{o}\right)=\exp\left(i\beta \;\left(x_{o}^{3}+y_{o}^{3}\right)\right)$. Thus, taking into account Equations (1) and (2), as an example, the transmittance of the generated microplate, with the design parameters summarized in Table 1, can be observed in Figure 1.

The electric field in the Cartesian coordinates solution to the Maxwell’s equations in the frequency domain for the region z>0 generated by the diffraction of an incident field on the designed microplate (Figure 1b) is given by:

(3) $$\vec{E}\left(x,y,z,w\right)=\vec{e}\left(x,y,z,\omega \right)\;F\left(\omega \right)$$

where F(ω) is obtained from the Fourier transform of the incident temporal pulse, and the field components, according to Luneburg’s vectorial diffraction theory $\vec{e}\left(x,y,z,\omega \right)$ [46], are:

(4) $$\begin{array}{ccc} e_{x}\left(x,y,z,\omega \right) & = & -\frac{z}{2\pi }\int \int_{\Sigma }E_{x}\left(x_{o},y_{o}\right)\;K_{L}\left(R\right)\;dx_{o}\;dy_{o}\\ e_{y}\left(x,y,z,\omega \right) & = & -\frac{z}{2\pi }\int \int_{\Sigma }E_{y}\left(x_{o},y_{o}\right)\;K_{L}\left(R\right)\;dx_{o}\;dy_{o}\\ e_{z}\left(x,y,z,\omega \right) & = & \frac{1}{2\pi }\int \int_{\Sigma }\left(\Delta_{x}E_{x}\left(x_{o},y_{o}\right)+\Delta_{y}E_{y}\left(x_{o},y_{o}\right)\right)\\ & & \;\times K_{L}\left(R\right)dx_{o}\;dy_{o} \end{array}$$

Σ being the BZP surface, $\Delta_{x}=\left(x-x_{o}\right)$, $\Delta_{y}=\left(y-y_{o}\right)$, $R={\left[\Delta_{x}^{2}+\Delta_{y}^{2}+z^{2}\right]}^{1/2}$, k=ωn/c is the wave number, *n* the refractive index of the medium and $K_{L}\left(R\right)=\left(i\;k\;R-1\right)\;R^{-3}\;\exp\left(i\;k\;R\right)$ is Luneburg’s kernel. Furthermore, $E_{x}\left(x_{o},y_{o}\right)$ and $E_{y}\left(x_{o},y_{o}\right)$ correspond to the fields generated by the zonal plate, which are related to the polarization state of the incident field ($E_{xo}$,$E_{yo}$) and also characterized by the designed binary transmittance $T_{ZM}$:

(5) $$\begin{array}{ccc} E_{x}\left(x_{o},y_{o}\right) & = & E_{xo}\;T_{ZM}\left(x_{o},y_{o}\right)\\ E_{y}\left(x_{o},y_{o}\right) & = & E_{yo}\;T_{ZM}\left(x_{o},y_{o}\right) \end{array}$$

In the same way, the corresponding magnetic field in the Cartesian coordinates solution to the Maxwell’s equations in the frequency domain for the region z>0 is given by:

(6) $$\vec{H}\left(x,y,z,w\right)=\vec{h}\left(x,y,z,\omega \right)\;F\left(\omega \right)$$

$\vec{h}\left(x,y,z,\omega \right)$ can be obtained through the Faraday’s law ($ik\eta \vec{h}\left(x,y,z,w\right)=\nabla \times \vec{e}\left(x,y,z,w\right)$) where η is the impedance in the propagation medium. Thus, the magnetic field components can obtained by introducing Equation (4) in Faraday’s law:

(7) $$\begin{array}{cc} h_{x}\left(x,y,z,\omega \right)=\frac{i}{2\pi k\eta }\int \int_{\Sigma } & ((\frac{\Delta_{x}\Delta_{y}E_{x}\left(x_{o},y_{o}\right)-\Delta_{x}^{2}E_{y}\left(x_{o},y_{o}\right)}{R^{2}})\left(3K_{L}\left(R\right)+K_{LM}\left(R\right)\right)\\ & +E_{y}\left(x_{o},y_{o}\right)\;\left(K_{L}\left(R\right)+K_{LM}\left(R\right)\right))dx_{o}\;dy_{o}\\ h_{y}\left(x,y,z,\omega \right)=-\frac{i}{2\pi k\eta }\int \int_{\Sigma } & ((\frac{\Delta_{x}\Delta_{y}E_{y}\left(x_{o},y_{o}\right)-\Delta_{y}^{2}E_{x}\left(x_{o},y_{o}\right)}{R^{2}})\left(3K_{L}\left(R\right)+K_{LM}\left(R\right)\right)\\ & +E_{x}\left(x_{o},y_{o}\right)\;\left(K_{L}\left(R\right)+K_{LM}\left(R\right)\right))dx_{o}\;dy_{o}\\ h_{z}\left(x,y,z,\omega \right)=-\frac{iz}{2\pi k\eta }\int \int_{\Sigma } & ((\frac{\Delta_{x}E_{y}\left(x_{o},y_{o}\right)-\Delta_{y}E_{x}\left(x_{o},y_{o}\right)}{R^{2}})\left(3K_{L}\left(R\right)+K_{LM}\left(R\right)\right))dx_{o}\;dy_{o} \end{array}$$

where $K_{LM}\left(R\right)=k^{2}\;R^{-1}\;\exp\left(i\;k\;R\right)$ is Luneburg’s magnetic kernel

In this work, we analyzed temporal Gaussian profiles, so we used a F(ω) function given by:

(8) $$F\left(\omega \right)=\exp\left(-b\;{\left(\omega -\omega_{0}\right)}^{2}\right)$$

where $\omega_{0}$ corresponds to the design frequency of the microplate Equation (2), and we used the parameter value $b=7.21\times 10^{-31}$ ((rad/s)${}^{-2}$), which corresponds to an initial temporal Gaussian pulse of δt=20 fs. Thus, the Airy–Gauss bullets were obtained by illuminating the microplate by an electric field with a temporal Gaussian profile. The numerical procedure is detailed as follows:

1. The microplate is illuminated by an incident circular electrical field given by a polarized spatial Gaussian wave ${\vec{E}}_{i}=\left(\hat{x}+i\hat{y}\right)\;e^{-\beta_{g}\left(x_{o}^{2}+y_{o}^{2}\right)}$ with $\beta_{g}=2\times 10^{9}$ (m${}^{-2}$).
2. The spectral bandwidth of the incident pulse, Δω, is discretized $\Delta \omega =\{\omega_{1},\omega_{2},\cdots \omega_{N}\}$
3. At each z-plane the spatial distributions of the non-diffracting beam ($\vec{e}\left(x,y,z,\omega_{i}\right)$ and $\vec{h}\left(x,y,z,\omega_{i}\right)$) is numerically obtained by introducing Equations (1) and (2) with the above-mentioned incident electrical field in Equation (4) (for the electrical field) and in Equation (7) (for the magnetic field).
4. For computational purposes, by taking into account the Convolution theorem, Equations (4) and (7) are numerically solved by means of fast Fourier transform operations by using [47,48]:
5. Finally, the Airy–Gauss bullets at each z plane are obtained by the discrete inverse Fourier transform by using the spatial distributions of the non-diffracting beam at each frequency ($\vec{e}\left(x,y,z,\omega_{i}\right)$ and $\vec{h}\left(x,y,z,\omega_{i}\right)$):
  (9) $$\begin{array}{c} \vec{E}\left(x,y,z,t_{K}\right)=\frac{1}{\sqrt{2\pi }}\sum_{i=1}^{N}\vec{e}\left(x,y,z,\omega_{i}\right)\;F\left(\omega_{i}\right)\exp\left(-i\omega_{i}t_{K}\right)\delta \omega \end{array}$$
  (10) $$\begin{array}{c} \vec{H}\left(x,y,z,t_{K}\right)=\frac{1}{\sqrt{2\pi }}\sum_{i=1}^{N}\vec{h}\left(x,y,z,\omega_{i}\right)\;F\left(\omega_{i}\right)\exp\left(-i\omega_{i}t_{K}\right)\delta \omega \end{array}$$

It is important to note that the validity of the numerical treatment used in this work is widely accepted as it has been compared to commercially available FDTD software [49,50,51]. Moreover, due to the dimensions of the proposed systems, the methodology used in this work presents significant advantages over 3D FDTD (accuracy, reliability, and computational cost).

## 3. Results and Discussion

First of all, we are analyzed the averaged total intensity at the studied temporal range at each plane (80 fs). To do this, in order to characterize the electromagnetic field distribution, we defined a normalized temporal averaged electromagnetic intensity to the maximum at focal plane as:

(11) $$I_{F}\left(x,y,z\right)=\frac{<|F_{x}{\left(x,y,z\right)|}^{2}+|F_{y}{\left(x,y,z\right)|}^{2}+{\left|F_{z}\left(x,y,z\right)\right|}^{2}>}{Max(|F_{x}\left(x,y,f\right){|}^{2}+|F_{y}\left(x,y,f\right){|}^{2}+|F_{z}\left(x,y,f\right){|}^{2})}$$

where F, *E*, or *H* are for electrical and magnetic fields, respectively. Moreover, each temporal averaged vectorial component can be obtained by using in the numerator of Equation (11) only the corresponding component.

**Figure 2:**
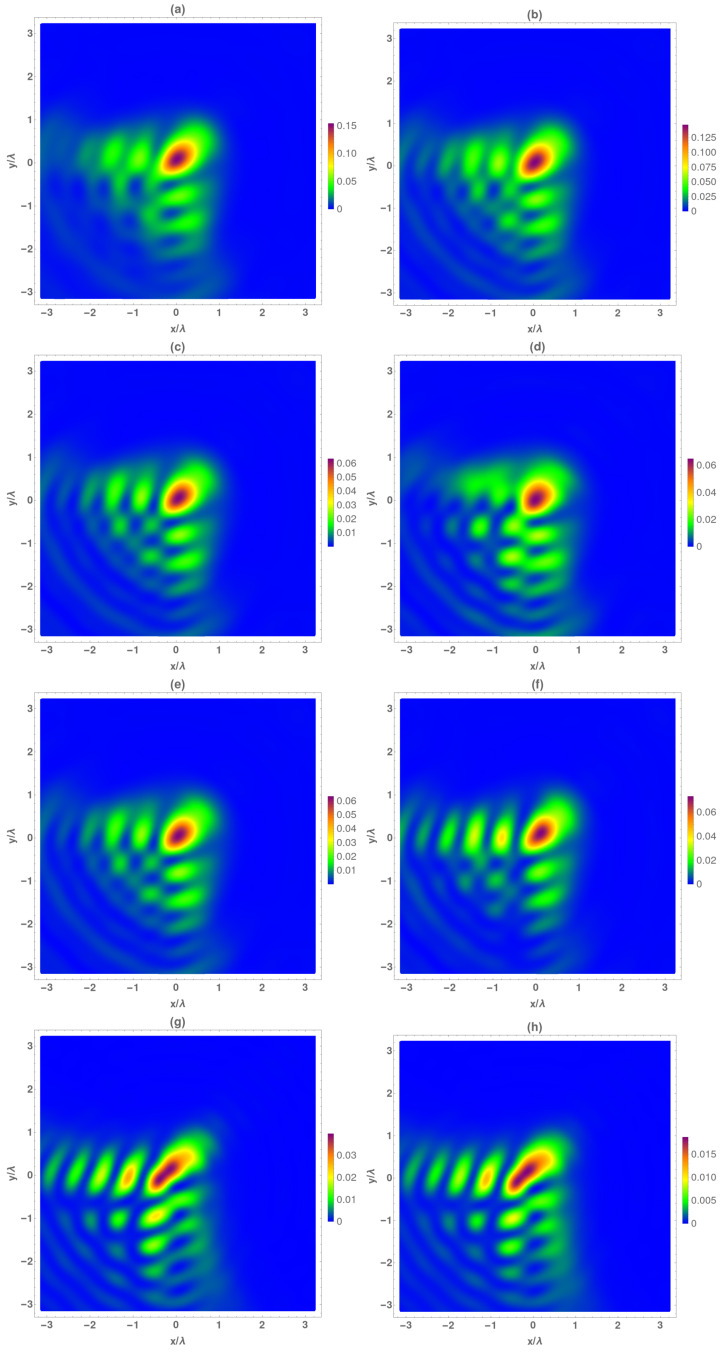
Temporal average of normalized electrical ($I_{E}$) and magnetic intensity ($I_{H}$) at the focal plane $z=f-2\lambda_{0}$. (**a**) $I_{E}$, (**b**) $I_{H}$, (**c**) $I_{Ex}$, (**d**) $I_{Hx}$, (**e**) $I_{Ey}$, (**f**) $I_{Hy}$, (**g**) $I_{Ez}$ and (**h**) $I_{Hz}$.

In Figure 2, we analyzed the electrical and magnetical temporal average intensity at $z=f-2\lambda_{0}$. As in all the cases, the averaged intensity was lower than the maximum value reached at the focal. At this plane, the Airy beam was not completely formed, but the total intensity of the main lobe was close to 15% of the maximum intensity reached. The transversal components (Figure 2c,e) are identical, (as can be deduced from Equation (4)), and both contribute to 80% of the total intensity (Figure 2a). The longitudinal component (Figure 2g) reached the 20% of the total intensity at this plane, presenting high deformation at the main lobe and at the side lobes as well. Moreover, by comparing to the transversal components, an offset in the positions of maximums of all the lobes appeared.

In a similar manner, following the same methodology, the corresponding magnetic field was analyzed at this figure. As it occurs with the electric field, the Airy beam is not totally formed at this plane, presenting lower-intensity values with respect to the maximum reached by the electrical field (Figure 2b). The main difference in the behavior of the electrical and magnetic field is given by the transversal components. As can be seen in Figure 2d,f, $H_{x}$ and $H_{y}$ are quite different, (as can be deduced from Equation (7)), mainly in the side lobes of both perpendicular directions; the $H_{x}$ component presents higher intensity at the *x* = 0 lobes whereas $H_{y}$ does at the *y* = 0 axis. The $H_{z}$ component is different to the others, but, in contrast, it is quite similar to $E_{z}$ (Figure 2g,h), with its contribution to the total intensity in this plane near 12%.

**Figure 3:**
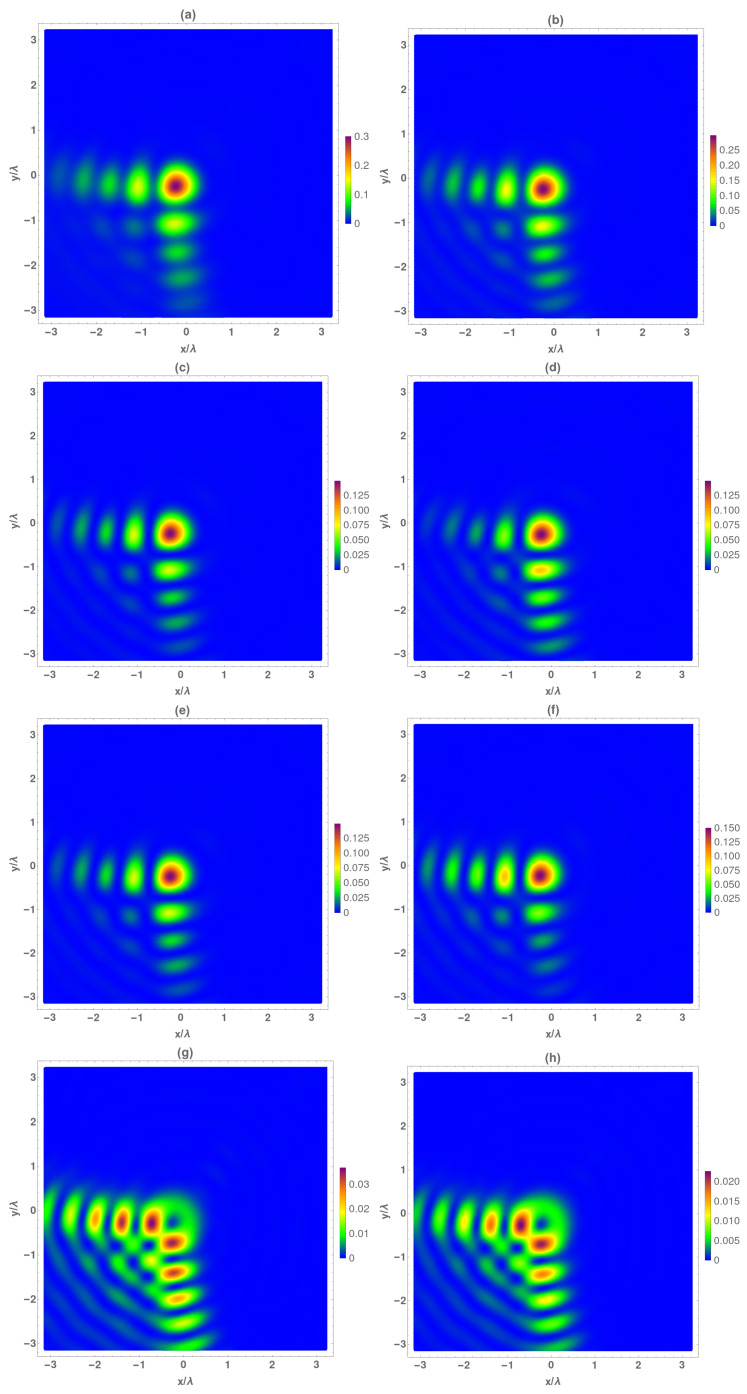
Temporal average of normalized electrical ($I_{E}$) and magnetic intensity ($I_{H}$) at the focal plane z=f. (**a**) $I_{E}$, (**b**) $I_{H}$, (**c**) $I_{Ex}$, (**d**) $I_{Hx}$, (**e**) $I_{Ey}$, (**f**) $I_{Hy}$, (**g**) $I_{Ez}$, and (**h**) $I_{Hz}$.

At the focal plane (Figure 3), the Airy beam is completely formed. The total electromagnetic intensity (Figure 3a,b) of the main lobe was raised, and the main corner lobes contained a large percentage of the beam’s total intensity. It is important to note that at the focal the main lobe of the temporal averaged electrical and magnetic Airy beam presented a subwavelength FWHM value. It is also worth mentioning that the raise (double with respect to $z=f-2\lambda_{0}$ plane) of the total electromagnetic intensity was mainly given by the corresponding amount of the transversal components one (Figure 3c–f). As previously described, the electric transversal components were quite similar among them, but the magnetic ones were different. At the focal plane, it can be clearly seen that the $H_{x}$ component presents higher intensity at the x=0 lobes, whereas $H_{y}$ does so at the y=0 axis. Finally, in relation to the intensity of $E_{z}$ and $H_{z}$, the values of the intensities are similar to the obtained at the $z=f-2\lambda_{0}$ plane (Figure 2g,h), but the intensity structure is too different to the other plane, because in this case both components resemble a Vortex–Airy beam [52].

This Vortex–Airy beam behavior can be explained by transforming the electromagnetic field given by Equations (4) and (7) to polar coordinates. Thus, taking into account that the incident field is circularly polarized and at plane z = 0 ($x_{o}=\rho \;cos\left(\theta \right),y_{o}=\rho \;sin\left(\theta \right)$) and at any z plane (x=rcos(ψ),y=r,sin(ψ)), the electric field given by Equation (4) is written as:

(12) $$\begin{array}{ccc} & & e_{x}\left(r,\psi ,z,\omega \right)=-\frac{z}{2\pi }\int_{0}^{a}\int_{0}^{2\pi }E_{x}\left(\rho ,\theta \right)K_{L}\left(R\right)\;\rho \;d\rho \;d\theta \\ & & e_{y}\left(r,\psi ,z,\omega \right)=\mp ie_{x}\left(r,\psi ,z\right)\\ & & e_{z}\left(r,\psi ,z,\omega \right)=\frac{1}{2\pi }\int_{0}^{a}\int_{0}^{2\pi }\Psi \left(r,\theta ,\rho ,\psi \right)K_{L}\left(R\right)\rho \;d\rho d\theta \end{array}$$

being in this case $R={\left[r^{2}+\rho^{2}+z^{2}-2r\rho \;cos\left(\theta -\psi \right)\right]}^{1/2}$, and the function Ψ is given by:

(13) $$\Psi \left(r,\theta ,\rho ,\psi \right)=E_{x}\left(\rho ,\theta \right)\left(r\;exp(\pm i\psi )-\rho \;exp(\pm i\theta )\right)$$

As it can be observed, the second term of function $\Psi (E_{x}\left(\rho ,\theta \right)exp\left(\pm i\theta \right))$ introduces a topological charge term ±1 to the generated fields by the zonal plate $E_{x}\left(\rho ,\theta \right)$, which results in a vortex beam behavior of $e_{z}$ component.

In the same way, the axial component of the magnetic field can be written as:

(14) $$h_{z}\left(r,\psi ,z,\omega \right)=-\frac{iz}{2\pi k\eta }\int_{0}^{a}\int_{0}^{2\pi }i\Psi \left(r,\theta ,\rho ,\psi \right)R^{-2}\left(3K_{L}\left(R\right)+K_{LM}\left(R\right)\right)\rho \;d\rho d\theta$$

Therefore, $h_{z}$ has the same topological charge as the axial component of the electric field and will present a vortex beam behavior.

**Figure 4:**
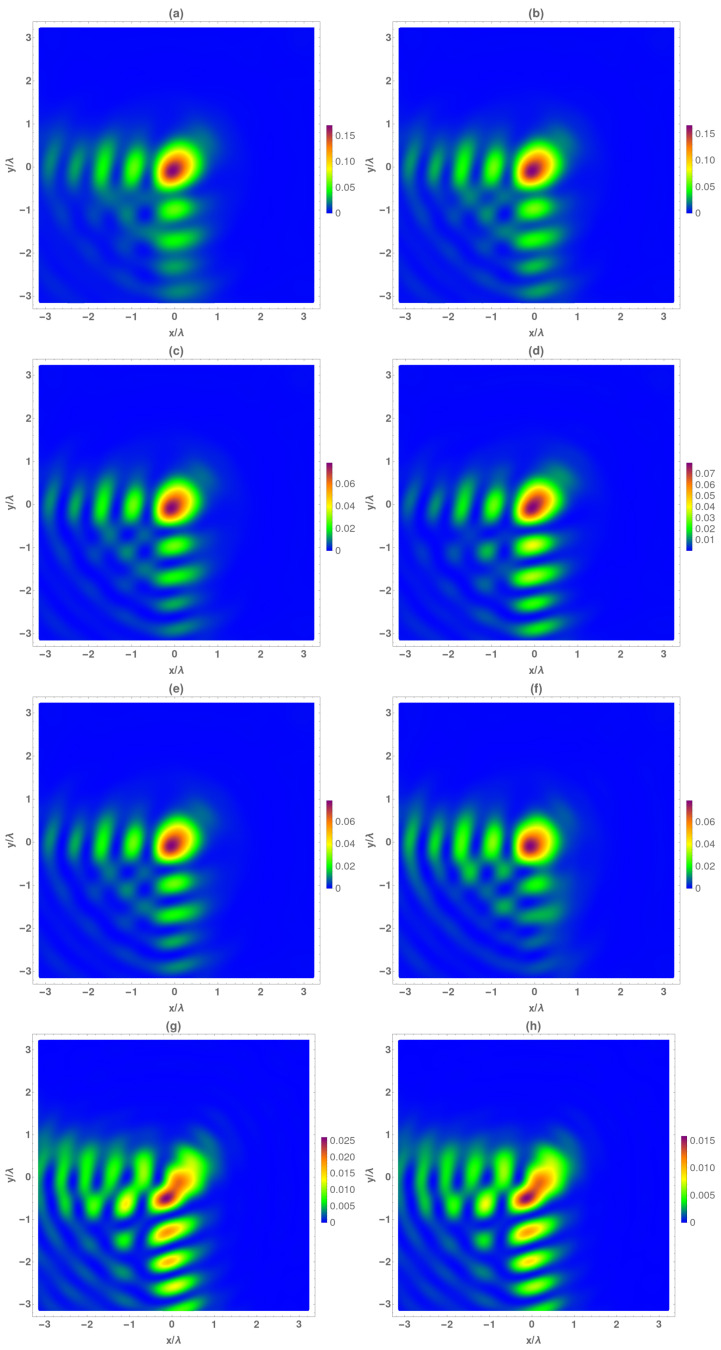
Temporal average of normalized electrical ($I_{E}$) and magnetic intensity ($I_{H}$) at the focal plane $z=f+2\lambda_{0}$. (**a**) $I_{E}$, (**b**) $I_{H}$, (**c**) $I_{Ex}$, (**d**) $I_{Hx}$, (**e**) $I_{Ey}$, (**f**) $I_{Hy}$, (**g**) $I_{Ez}$, and (**h**) $I_{Hz}$.

Finally, the temporal average intensity of the electric and magnetic vectorial components are analyzed in Figure 4 at $z=f+2\lambda_{0}$ plane. As can be seen, the behavior is not symmetrical with respect to the focal plane, with the structure of the Airy beam being maintained, but, the main lobe began to deform. The value of the total intensity decreased with respect to the focal, as before, mainly given by the transversal components. Regarding, transversal field components, the behavior was the same as above; the electrical components were quite similar among them, and the $H_{x}$ component presented higher intensity at the x = 0 lobes whereas $H_{y}$ did at the y = 0 axis. It is important to remark that, in contrast to the observed behavior at $z=f-2\lambda_{0}$, the structure form of these components corresponds to a lightly deformed Airy beam. However, the longitudinal components are quite similar between them and also to the obtained at $z=f-2\lambda_{0}$, in both intensity and structure form.

**Figure 5:**
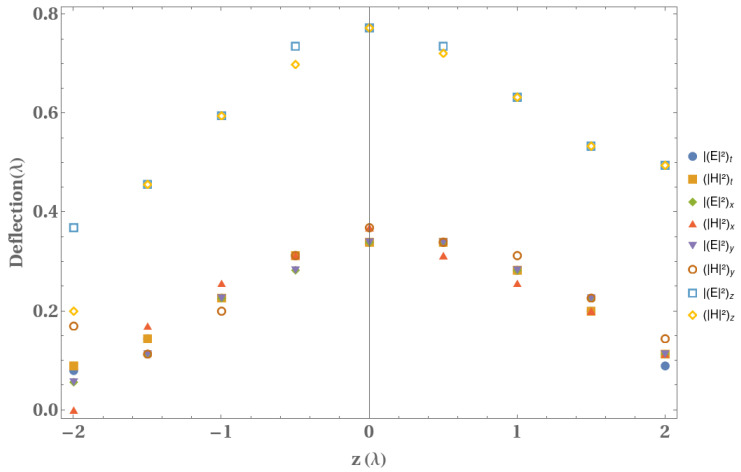
Variation of the deflection of the electrical and magnetic fields with the propagation coordinate for all the components.

In relation to this analysis, from the normalized intensities of the electrical and magnetic fields, in Figure 5 we show the deflection along the propagation distance for all the field components. A parabolic trajectory can be clearly seen for both field and each component, which agree to the previous theoretical descriptions [17,53]. Moreover, due to the Airy vortex beam’s similar behavior of the axial components ($E_{z}$ and $H_{z}$), the deflection is larger.

**Figure 6:**
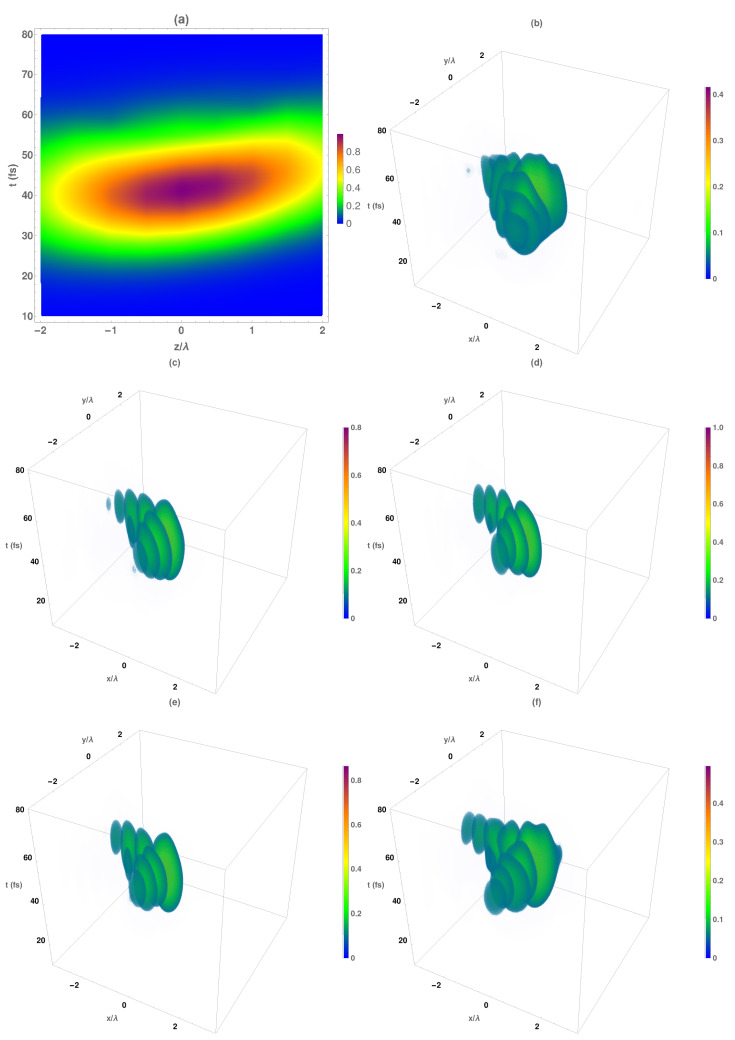
(**a**) Temporal evolution of the magnetic normalized maximum intensity with respect to the focal plane of the obtained Airy–Gauss bullets obtained by using the designed BZP. Temporal evolution of the normalized total intensity $I_{H}$ patterns at several z planes: (**b**) $z=f-2\lambda_{0}$, (**c**) $z=f-\lambda_{0}$, (**d**) focal plane (z=f), (**e**) $z=f+\lambda_{0}$, (**f**) $z=f+2\lambda_{0}$.

Once we performed the analysis of the average intensities, we analyzed the corresponding Airy–Gauss vectorial bullets obtained by the proposed methodology described above. At this point, the magnetic intensity was studied, but similar results were obtained with the electrical one. In Figure 6a, we present the temporal variation of the maximum magnetic intensity at each propagation distance. As can be seen, the maximum intensity of the temporal Gaussian pulse was approximately located at a range of $\pm \lambda_{0}$ from the focal plane and obtained for times t∼40 fs, a value that was delayed by diffraction effects with respect to the expected time of $\frac{fn}{c}=35$ fs. Moreover, 50% of the peak intensity can be obtained in the range between −1 to 2 $\lambda_{0}$.

The temporal evolution of the intensity distributions of the Airy–Gauss bullets at several z planes is shown in Figure 6b–f. As mentioned above, the asymmetrical behavior with respect to the focal plane is clearly demonstrated. At a distance $z=f-2\lambda_{0}$ (Figure 6b), the spatial structure of the Airy beam appeared with deformed low-intensity secondary lobes, and the main temporal-spatial lobe reached intensity values of 40%. Near the focal plane (Figure 6c), the spatial Airy beam was formed (presenting a well-defined ellipsoid form for all the lobes) with intensity close to 80% at the main spatial lobe. At the focal plane (Figure 6d), the maximum intensity value was reached at the main temporal-spatial lobe. Both secondary spatio-temporal peaks contained around 50% of the power, the spatial Airy being maintained up to 50 fs. Finally, at longer distances from the focal plane, (Figure 6f), the intensity of the central and secondary lobes decreased, but their values were higher than the corresponding values at z<f. Moreover, at $z=f+2\lambda_{0}$ (Figure 6f), deformations in the spatio-temporal ellipsoidal structures of all the lobes began.

**Figure 7:**
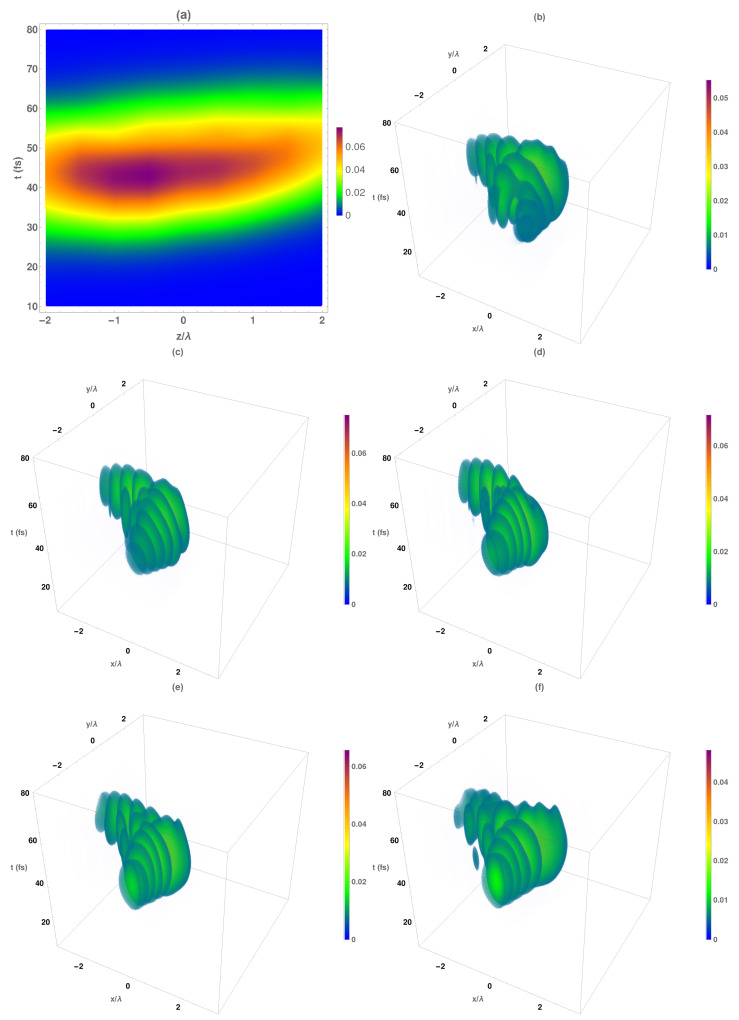
(**a**) Temporal evolution of the longitudinal magnetic normalized maximum intensity with respect to the focal plane of the obtained Airy–Gauss bullets obtained by using the designed BZP. Temporal evolution of the normalized total intensity $I_{Hz}$ patterns at several z planes: (**b**) $z=f-2\lambda_{0}$, (**c**) $z=f-\lambda_{0}$, (**d**) focal plane (z=f), (**e**) $z=f+\lambda_{0}$, (**f**) $z=f+2\lambda_{0}$.

As we stated above, the transverse components of the electromagnetic field present the same spatio-temporal structure as the total intensity shown in Figure 6. Moreover, the intensity change along the propagation direction is mainly produced by these components. However, the behavior of the electromagnetic longitudinal components is different to the transversal ones. Due to the fact that $H_{z}$ component response is similar to the corresponding electrical one, we analyzed in Figure 7 the longitudinal magnetic intensity of the Airy–Gauss bullets. In Figure 7a, the temporal variation in the maximum magnetic intensity at each propagation distance is shown. As can be seen, the maximum intensity of the temporal Gaussian pulse was approximately located at all the analyzed range being the higher values obtained at z<f.

The corresponding temporal evolution of the intensity distribution of the longitudinal magnetic component of the Airy–Gauss bullets at several z planes is shown in Figure 7b–f. At a distance $z=f-2\lambda_{0}$ (Figure 7b), the spatial structure of the Airy beam was not formed (both main and secondary lobes). Near to the focal plane (Figure 7c), the spatial Airy beam was formed, presenting the secondary lobes a well-defined ellipsoid form with a deformed main lobe that presents null intensity similar to an Airy vortex beam. Finally, at longer distances from the focal plane, (Figure 7f), the ellipsoidal form of the secondary lobes was maintained, but the main lobe was deformed.

Finally, taking the advantage of this vectorial analysis, we analyzed several magnitudes such as the Poynting vector, the energy, and the Helicity of the Airy–Gauss bullets at each plane. In this sense, the temporal averaged values of these magnitudes were calculated by:

(15) $$G\left(x,y,z\right)=\frac{1}{2\pi }\sum_{i=1}^{N}G\left(x,y,z,\omega_{i}\right)\delta \omega$$

where G corresponds to $\vec{S}$ (Poynting vector); *U* (total energy) and H (helicity). Thus, $\vec{S}\left(x,y,z,\omega_{i}\right)$ was calculated from the electromagnetic fields $\vec{e}\left(x,y,z,\omega_{i}\right)$ and $\vec{h}\left(x,y,z,\omega_{i}\right)$ obtained from Equations (4) and (7) as:

(16) $$\vec{S}\left(x,y,z,\omega_{i}\right)=\frac{1}{2}ℜ\left\{\vec{e}\left(x,y,z,\omega_{i}\right)\times {\vec{h}}^{*}\left(x,y,z,\omega_{i}\right)\right\}$$

The total energy was calculated according to

(17) $$U\left(x,y,z,\omega_{i}\right)=\frac{1}{4}\left(\epsilon \vec{e}\left(x,y,z,\omega_{i}\right)\cdot {\vec{e}}^{*}\left(x,y,z,\omega_{i}\right)+\mu \vec{h}\left(x,y,z,\omega_{i}\right)\cdot {\vec{h}}^{*}\left(x,y,z,\omega_{i}\right)\right)$$

where ϵ and μ correspond to the electrical and magnetic permittivity, respectively, of the propagation media.

**Figure 8:**
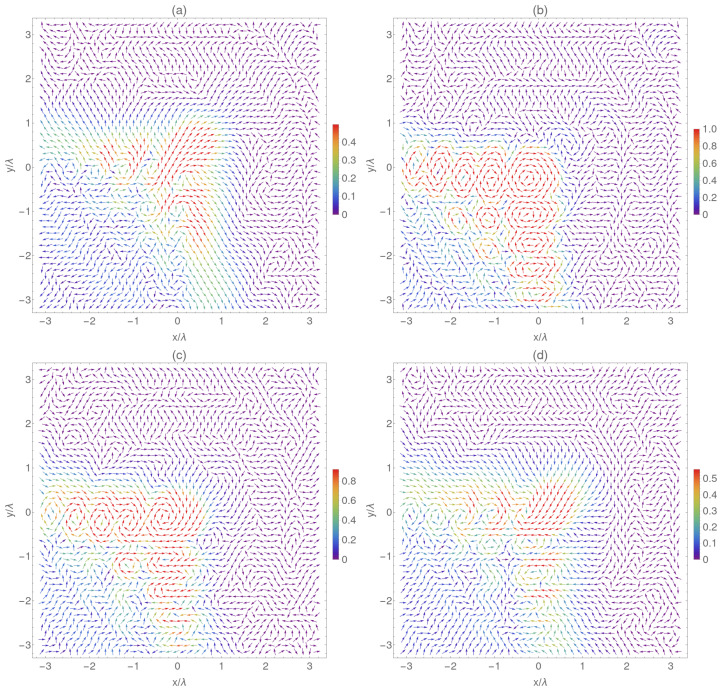
Temporal averaged normalized transversal Poynting vector respect to the maximal norm at the focal plane. (**a**) $z=f-2\lambda_{0}$, (**b**) focal plane (z=f), (**c**) $z=f+\lambda_{0}$, (**d**) $z=f+2\lambda_{0}$.

For the analysis of the Poynting vector in Airy–Gauss bullets, in Figure 8 we show the temporal averaged transversal components at different propagation distances. It is important to note that the longitudinal component is an order of magnitude higher than these ones, but, we centered our study on the transversal components. As it can be seen, the magnitude of the transversal Poynting vector was higher at the lobes, where a circulation related to polarization state was observed. Among the circulation at the main lobes, at $z=f-2\lambda_{0}$, there was a preferential direction of 45${}^{\circ }$ pointing from the main lobe with positive slope (sense where the auto-acceleration is produced). At the focal, there was no preferential direction of the net energy flow, but the magnitude was the highest one, and a significant circulation was observed around the main and secondary lobes of the Airy structure. At z>f, the energetic circulation around the main lobes was maintained, but a preferential direction of 45${}^{\circ }$ appeared pointed in the opposite direction than at z<f (towards the main lobe), being the magnitude of the transversal Poynting vector higher at shorter distances from the focal.

**Figure 9:**
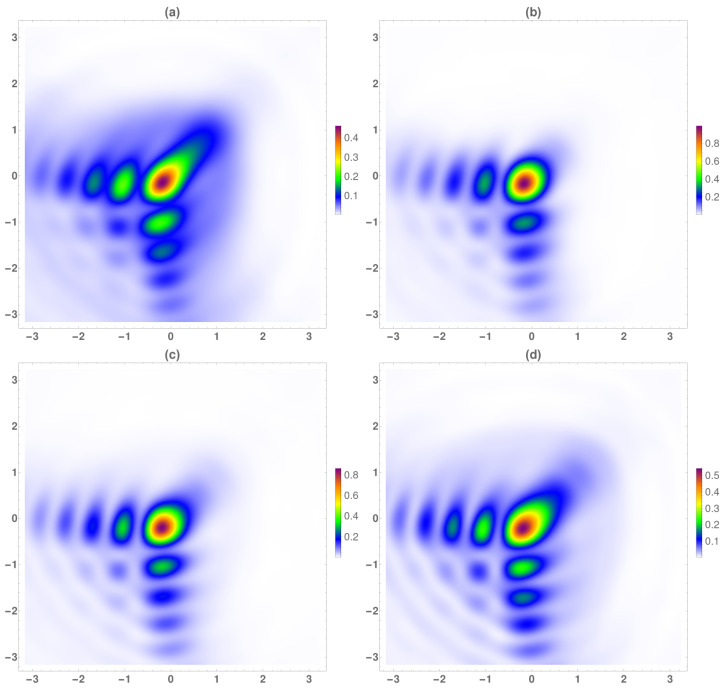
Temporal averaged normalized energy respect to the maximum value at the focal plane. (**a**) $z=f-2\lambda_{0}$, (**b**) focal plane (z=f), (**c**) $z=f+\lambda_{0}$, (**d**) $z=f+2\lambda_{0}$.

Regarding to the energy, in Figure 9, the temporal averaged normalized energy with respect to the maximum value at the focal plane was analyzed. The value of the energy was maximum at the focal plane, as can be seen in Figure 9a, at $z=f-2\lambda_{0}$, the difference among the secondary to the main corners lobes was around the 50%. At the focal, the highest energy peak was at the main lobe, the difference between the main corners lobes being higher than at z<f. Finally, at longer distances from the focal, the energy decreased as well the difference between the main and secondary lobes.

Finally, together with energy, helicity is an important property of light, although it is closely related to its spin, and the polarization of light. In this sense, we obtained that this magnitude conserves along the propagation direction.

## 4. Conclusions

In conclusion, based on the vectorial diffraction theory we presented a methodology for obtaining and analyzing vectorial non-diffracting bullets. Thus, we proposed a design methodology to obtain binary zonal microplates (BZP) for generating Airy–Gauss bullets. For near field applications, based on an exact solution to Maxwell’s equations, vectorial Airy–Gauss bullets were analyzed, developing a methodology for the study of the corresponding magnetic field. In this article, we presented circular polarized Airy–Gauss bullets generated from Gaussian temporal pulses of 20 fs obtained by the designed microplate of high numerical aperture, resulting in Airy–Gauss bullets with a FWHM close to 0.75 $\lambda_{0}$. A complete analysis of the electromagnetic propagation was performed, paying special attention to the magnetic components (both transversal and longitudinal). It was observed that the intensity variation in the Airy–Gauss bullets was mainly given by the transversal components and that the contribution of the longitudinal one was maintained along the propagation distance. Taking into account the complete electromagnetic study, several magnitudes were obtained, such as the temporal averaged Poynting vector and the energy of the generated bullets. The transverse temporal averaged Poynting vector presents a circulation in the Airy lobes, the highest magnitude being obtained at the focal.

## Figures and Tables

**Table 1:** Design parameters of the binary micro zonal plate shown in Figure 1a.

Parameters	Values
$\lambda_{0}$	532 nm
β	$6\times 10^{15}$ (m${}^{-3}$)
*f*	15 ($\lambda_{0}$)
n	1.33
Dimensions	$40\times 40(\lambda_{0}^{2})$

## References

[1] Christodoulides D., Wengerowsky S., Rao S.M. (2016). Optical airy beams and bullets. Front. Mod. Opt..

[2] Polynkin P., Kolesik M., Moloney J.V., Siviloglou G.A., Christodoulides D.N. (2009). Curved Plasma Channel Generation Using Ultraintense Airy Beams. Science.

[3] Minovich A.E., Klein A.E., Neshev D.N., Pertsch T., Kivshar Y.S., Christodoulides D.N. (2014). Airy plasmons: Non-diffracting optical surface waves. Laser Photonics Rev..

[4] Liang Y., Hu Y., Song D., Lou C., Zhang X., Chen Z., Xu J. (2015). Image signal transmission with Airy beams. Opt. Lett..

[5] Baumgartl J., Mazilu M., Dholakia K. (2008). Optically mediated particle clearing using Airy wavepackets. Nat. Photonics.

[6] Abdollahpour D., Suntsov S., Papazoglou D.G., Tzortzakis S. (2010). Spatiotemporal Airy Light Bullets in the Linear and Nonlinear Regimes. Phys. Rev. Lett..

[7] Christodoulides D.N. (2008). Optical Trapping Riding along an Airy beam. Nat. Photonics.

[8] Suarez R.A.B., Neves A.A.R., Gesualdi M.R.R. (2021). Optical trapping with non-diffracting Airy beams array using a holographic optical tweezers. Opt. Laser Technol..

[9] Nylk J., McCluskey K., Aggarwal S., Tello J.A., Dholakia K. (2016). Enhancement of image quality and imaging depth with Airy light-sheet microscopy in cleared and non-cleared neural tissue. Biomed. Opt. Express.

[10] Dholakia K. (2019). New Perspectives for Biomedical Imaging at Depth. Biophotonics Australas..

[11] Ren Y.X., He H., Tang H., Wong K.K.Y. (2021). Non-Diffracting Light Wave: Fundamentals and Biomedical Applications. Front. Phys.-Lausanne.

[12] Siviloglou G.A., Christodoulides D.N. (2007). Accelerating finite energy Airy beams. Opt. Lett..

[13] Siviloglou G.A., Broky J., Dogariu A., Christodoulides D.N. (2007). Observation of accelerating airy beams. Phys. Rev. Lett..

[14] Dai H.T., Sun X.W., Luo D., Liu Y.J. (2009). Airy beams generated by a binary phase element made of polymer-dispersed liquid crystals. Opt. Express.

[15] Luo D., Dai H.T., Sun X.W. (2013). Polarization-independent electrically tunable/switchable Airy beam based on polymer-stabilized blue phase liquid crystal. Opt. Express.

[16] Cao R., Yang Y., Wang J., Bu J., Wang M., Yuan X.C. (2011). Microfabricated continuous cubic phase plate induced Airy beams for optical manipulation with high power efficiency. Appl. Phys. Lett..

[17] Cai Z., Liu Y., Zhang C., Xu J., Ji S., Ni J., Li J., Hu Y., Wu D., Chu J. (2017). Continuous cubic phase microplates for generating high-quality Airy beams with strong deflection. Opt. Lett..

[18] Cai W., Wang L., Wen S. (2018). Role of third-order dispersion in chirped Airy pulse propagation in single-mode fibers. Opt. Commun..

[19] Nanfang Y., Patrice G., Kats Mikhail A., Francesco A., Jean-Philippe T., Federico C., Zeno G. (2011). Light Propagation with Phase Discontinuities: Generalized Laws of Reflection and Refraction. Science.

[20] Guo W.L., Chen K., Wang G.M., Luo X.Y., Cai T., Zhang C.B., Feng Y. (2020). Airy Beam Generation: Approaching Ideal Efficiency and Ultra Wideband with Reflective and Transmissive Metasurfaces. Adv. Opt. Mater..

[21] Song E.Y., Lee G.Y., Park H., Lee K., Kim J., Hong J., Kim H., Lee B. (2017). Compact Generation of Airy Beams with C-Aperture Metasurface. Adv. Opt. Mater..

[22] Wen J., Chen L., Yu B., Nieder J.B., Zhuang S., Zhang D., Lei D. (2021). All-Dielectric Synthetic-Phase Metasurfaces Generating Practical Airy Beams. ACS Nano.

[23] Zhao Z., Ding X., Zhang K., Fu J., Burokur S.N., Wu Q. (2021). Generation and deflection control of a 2D Airy beam utilizing metasurfaces. Opt. Lett..

[24] Chremmos I., Efremidis N.K., Christodoulides D.N. (2011). Pre-engineered abruptly autofocusing beams. Opt. Lett..

[25] Papazoglou D.G., Efremidis N.K., Christodoulides D.N., Tzortzakis S. (2011). Observation of abruptly autofocusing waves. Opt. Lett..

[26] Li N., Jiang Y., Huang K., Lu X. (2014). Abruptly autofocusing property of blocked circular Airy beams. Opt. Express.

[27] Jiang Y., Zhu X., Yu W., Shao H., Zheng W., Lu X. (2015). Propagation characteristics of the modified circular Airy beam. Opt. Express.

[28] Liu Z., He Y., Sun Q., Ning Y., Xu X. (2021). Comparison of focusability between traditional beams and novel beams. Optik.

[29] Jiang Y., Cao Z., Shao H., Zheng W., Zeng B., Lu X. (2016). Trapping two types of particles by modified circular Airy beams. Opt. Express.

[30] Porfirev A.P. (2021). Laser manipulation of airborne microparticles behind non-transparent obstacles with the help of circular Airy beams. Appl. Opt..

[31] Lu F., Tan L., Tan Z., Wu H., Liang Y. (2021). Dynamical power flow and trapping-force properties of two-dimensional Airy-beam superpositions. Phys. Rev. A.

[32] Mitri F.G. (2021). Circularly-polarized Airy light-sheet spinner tweezers and particle transport. J. Quant. Spectrosc. Radiat..

[33] Khonina S.N., Porfirev A.P., Ustinov A.V. (2018). Sudden autofocusing of superlinear chirp beams. J. Opt..

[34] Porfirev A.P., Fomchenkov S.A., Khonina S.N. Experimental investigation of complex circular Airy beam characteristics. Proceedings of the Saratov Fall Meeting 2017: Laser Physics and Photonics Xviii; and Computational Biophysics and Analysis of Biomedical Data Iv.

[35] Berry M.V., Balazs N.L. (1979). Non-spreading Wave Packets. Am. J. Phys..

[36] Bongiovanni D., Wetzel B., Hu Y., Chen Z., Morandotti R. (2016). Optimal compression and energy confinement of optical Airy bullets. Opt. Express.

[37] Chong A., Renninger W.H., Wise F.W. Linear light bullets based on Airy-Bessel wave packets. Proceedings of the Nonlinear Frequency Generation and Conversion: Materials, Devices, and Applications X.

[38] Li H., Huang X., Cao Q., Zhao Y., Li P., Wan C., Chong A. (2017). Generation of three-dimensional versatile vortex linear light bullets. Chin. Opt. Lett..

[39] Chong A., Renninger W.H., Christodoulides D.N., Wise F.W. (2010). Airy-Bessel wave packets as versatile linear light bullets. Nat. Photonics.

[40] Zhong W.P., Belic M.R., Huang T. (2013). Three-dimensional finite-energy Airy self-accelerating parabolic-cylinder light bullets. Phys. Rev. A.

[41] Deng F., Hong W. (2016). Chirp-Induced Channel of an Airy Pulse in an Optical Fiber Close to Its Zero-Dispersion Point. IEEE Photonics J..

[42] Zhong W.P., Belic M.R., Zhang Y. (2016). Airy-Tricomi-Gaussian compressed light bullets. Eur. Phys. J. Plus..

[43] Blaya S., Carretero L., Acebal P. (2020). Vectorial analysis of Airy-Airy bullets generated by high aperture binary micro zonal plate. Opt. Laser. Eng..

[44] Wu D., Qi X., Cai Z., Wang D., Hu Y., Li J., Chu J. (2021). Direct Generation of Airy Beams at Designed Fourier Planes Using Integrated Airy Phase Plates. IEEE Photonic Technol. Lett..

[45] Liu H., Lu Y., Zhang J., Xia J., Pu X., Dong Y., Li S., Fu X., Zhang A., Wang C. (2015). Research on propagation properties of controllable hollow flat-topped beams in turbulent atmosphere based on ABCD matrix. Opt. Commun..

[46] Luneburg R.K. (1964). Mathematical Theory of Optics.

[47] Shen F., Wang A. (2006). Fast-Fourier-transform based numerical integration method for the Rayleigh-Sommerfeld diffraction formula. Appl. Opt..

[48] Cottrell D.M., Davis J.A., Berg C.A., Freeman C.L. (2014). Analysis of the propagation dynamics and Gouy phase of Airy beams using the fast Fresnel transform algorithm. Appl. Opt..

[49] Ye H., Qiu C.W., Huang K., Teng J., Luk’yanchuk B., Yeo S.P. (2013). Creation of a longitudinally polarized subwavelength hotspot with an ultra-thin planar lens: Vectorial Rayleigh–Sommerfeld method. Laser Phys. Lett..

[50] Khonina S.N., Ustinov A.V., Kovalyov A.A., Volotovsky S.G. (2014). Near-field propagation of vortex beams: Models and computation algorithms. Opt. Mem. Neural Netw..

[51] Acebal P., Carretero L., Blaya S. (2021). Extraordinary spin to orbital angular momentum conversion on guided zone plates. Sci. Rep..

[52] Wei B.Y., Liu S., Chen P., Qi S.X., Zhang Y., Hu W., Lu Y.Q., Zhao J.L. (2018). Vortex Airy beams directly generated via liquid crystal q-Airy-plates. Appl. Phys. Lett..

[53] Polynkin P., Kolesik M., Moloney J. (2009). Filamentation of Femtosecond Laser Airy Beams in Water. Phys. Rev. Lett..

